# QuickStats

**Published:** 2013-06-07

**Authors:** Anjali Talwalkar, Jill J. Ashman

**Figure:**
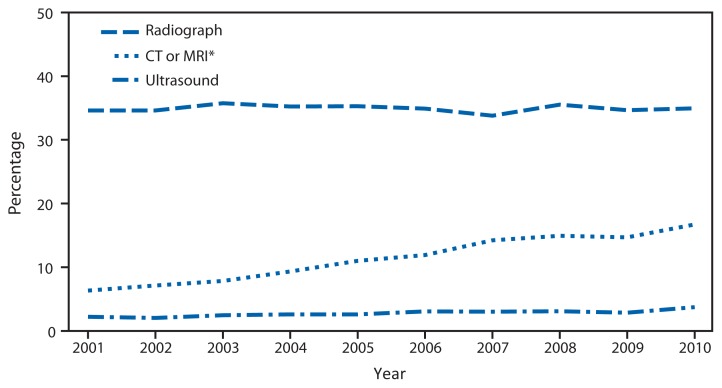
Annual Percentage of Emergency Department Visits with Selected Imaging Tests Ordered or Provided — National Hospital Ambulatory Medical Care Survey, United States, 2001–2010 **Abbreviations:** CT = computed tomography; MRI = magnetic resonance imaging. * Separate estimates for MRIs and CTs are available only for the period 2005–2010. During that period, visits with only an MRI accounted for <4% of the combined CT/MRI category.

From 2001 to 2010, the percentage of emergency department visits with a CT or MRI test ordered or provided nearly tripled from 6% to 17%, and the percentage of visits with an ultrasound ordered or provided doubled from 2% to 4%. The percentage of emergency department visits with a radiograph ordered or provided did not change significantly. Throughout the period, the percentage of visits with a radiograph was higher than the percentage with a CT/MRI or ultrasound combined and remained steady at about 35%.

**Source:** CDC. National Hospital Ambulatory Medical Care Survey. Available at http://www.cdc.gov/nchs/ahcd/ahcd_questionnaires.htm.

